# Inflammasome induction in Rasmussen’s encephalitis: cortical and associated white matter pathogenesis

**DOI:** 10.1186/1742-2094-10-152

**Published:** 2013-12-13

**Authors:** Vijay Ramaswamy, John G Walsh, D Barry Sinclair, Edward Johnson, Richard Tang-Wai, B Matt Wheatley, William Branton, Ferdinand Maingat, Thomas Snyder, Donald W Gross, Christopher Power

**Affiliations:** 1 Division of Pediatric Neurology, University of Alberta, Edmonton, AB, Canada; 2 Department of Medicine (Neurology), University of Alberta, Edmonton, AB, Canada; 3 Department of Laboratory Medicine and Pathology (Neuropathology), University of Alberta, Edmonton, AB, Canada; 4 Department of Surgery (Neurosurgery), University of Alberta, Edmonton, AB, Canada; 5 Department of Psychiatry, University of Alberta, Edmonton, AB, Canada; 6 Arthur and Sonia Labatt Brain Tumor Research Centre, Hospital for Sick Children, Toronto, ON, Canada; 7 Department of Medicine, University of Alberta, 6-11 Heritage Medical Research Centre, Edmonton, AB T6G 2S2, Canada

**Keywords:** Rasmussen’s encephalitis, White matter, Inflammasome, Innate immunity

## Abstract

**Background:**

Rasmussen’s encephalitis (RE) is an inflammatory encephalopathy of unknown cause defined by seizures with progressive neurological disabilities. Herein, the pathogenesis of RE was investigated focusing on inflammasome activation in the brain.

**Methods:**

Patients with RE at the University of Alberta, Edmonton, AB, Canada, were identified and analyzed by neuroimaging, neuropsychological, molecular, and pathological tools. Primary human microglia, astrocytes, and neurons were examined using RT-PCR, enzyme-linked immunosorbent assay (ELISA), and western blotting.

**Results:**

Four patients with RE were identified at the University of Alberta. Magnetic resonance imaging (MRI) disclosed increased signal intensities in cerebral white matter adjacent to cortical lesions of RE patients, accompanied by a decline in neurocognitive processing speed (*P* <0.05). *CD3ϵ*, *HLA-DRA*, and *TNFα* together with several inflammasome-associated genes (*IL-1β*, *IL-18, NLRP1*, *NLRP3*, and *CASP1)* showed increased transcript levels in RE brains compared to non-RE controls (n = 6; *P* <0.05). Cultured human microglia displayed expression of inflammasome-associated genes and responded to inflammasome activators by releasing IL-1β, which was inhibited by the caspase inhibitor, zVAD-fmk. Major histocompatibility complex (MHC) class II, IL-1β, caspase-1, and alanine/serine/cysteine (ASC) immunoreactivity were increased in RE brain tissues, especially in white matter myeloid cells, in conjunction with mononuclear cell infiltration and gliosis. Neuroinflammation in RE brains was present in both white matter and adjacent cortex with associated induction of inflammasome components, which was correlated with neuroimaging and neuropsychological deficits.

**Conclusion:**

Inflammasome activation likely contributes to the disease process underlying RE and offers a mechanistic target for future therapeutic interventions.

## Background

Rasmussen’s encephalitis (RE) is a rare progressive encephalopathy defined by chronic focal epilepsy, neurocognitive, and motor deficits with neuroinflammation [1,2]. Clinical features of RE include intractable seizures, hemiparesis, blindness, and neurocognitive dysfunction [3]. The major seizure semiology associated with RE is epilepsia partialis continua, which is difficult to control with current anti-epileptic drugs [4]. RE is chiefly a disease of children but occasionally occurs in adulthood (10%) and can be fatal depending on the severity of the disease. Treatment options for RE are limited to controlling the seizures with anti-epileptic drugs together with radical neurosurgical interventions (corticectomy or hemispherectomy) associated with major postoperative neurologic deficits including hemiparesis and hemianopsia [5]. Therapeutic immunomodulation and/or immunoablation in RE as well as antiviral therapies have been reported with inconclusive results [6,7].

Although there is substantial clinical heterogeneity, RE usually displays neuropathological features consisting of inflammation, gliosis, and tissue destruction in the cortex of the affected hemisphere, often with relative sparing of the basal ganglia, contralateral hemisphere, and posterior fossa structures. The observed inflammatory changes in RE include perivascular cuffing, microglial nodules, T lymphocytic infiltration gliosis, meningeal inflammation, and neuronal injury or loss [2,8]. The underlying etiology of RE is unknown; several exogenous pathogens have been postulated as causative [9] but autoimmune mechanisms have also been reported, largely based on the infiltration of T lymphocytes (CD8+) and macrophages at the disease onset accompanied by gliosis and neurodegeneration in the cerebral cortex [10].

Inflammation in the nervous system occurs in multiple neurological disorders with diverse causes and pathological phenotypes. Recently, the contribution of innate immunity to neurological disease has garnered increased attention, especially with the recognition of inflammasome activation [11]. Inflammasomes represent complex protein aggregates formed by cytosolic receptors, their adaptor proteins, and caspase-1, which regulate the maturation and release of IL-1β and IL-18 [12]. Activation of an inflammasome involves an orchestrated engagement of cell signaling molecules that primes cells to express pro-IL-1β (signal 1) with subsequent signals that trigger the formation of an inflammasome complex (signal 2). The formation of these complexes mediates the cleavage and release of active IL-1β, which participates in the development and promulgation of inflammatory responses. Inflammasome activation has recently been implicated in bacterial meningitis, autoimmune demyelination, and chronic neurodegenerative disease [13-15]. Nonetheless, little is known about inflammasome expression and function in human neural cells.

Given the disabling nature of the motor features associated with RE together with previous imaging and neuropathological studies suggesting white matter involvement, we hypothesized that the disease process extended beyond the cortex into the proximal white matter in RE and might engage inflammasome cellular components because of RE’s inflammatory features. In fact, the present studies revealed that among RE patients there was converging evidence of white matter pathogenesis, based on neuropsychological, neuroimaging, molecular, and neuropathological analyses. Moreover, induction of multiple components implicated in inflammasome expression was evident in both cortex and white matter of RE patients, particularly in activated myeloid cells.

## Methods and materials

### Clinical studies

A retrospective review of all patients diagnosed with RE in the Comprehensive Epilepsy Program was performed based on the published criteria for RE [4], using the demographic, clinical, laboratory, neuroimaging, and treatment data of patients followed in this program, with University of Alberta (Edmonton, AB, Canada) Ethics Committee approval. To confirm, corroborate, and establish the diagnosis of RE, all patients referred to the program with epilepsy and an abnormal cranial magnetic resonance imaging (MRI) were evaluated by a pediatric epileptologist (DBS, RTW). The patients presented herein (n = 4; ages 3 to 14 years) represent all identified cases of RE during the years from 1999 to 2009 (Table 1). Patient control brain specimens (mean age 19.4 ± 12.9 years; males, n = 4; mesial temporal sclerosis (n = 5), multiple sclerosis (n = 1)) for the molecular studies included both cortex and white matter.

**Table 1:** Clinical features of RE patients

	**Patient 1**	**Patient 2**	**Patient 3**	**Patient 4**
Age of onset, gender	3 years, female	8 years, female	14 years, male	12 years, female
Seizure type	CPS, Gen, right face/hand EPC	CPS, right face/hand EPC	CPS, right face/hand EPC	CPS, right face/hand EPC
Hemisphere	Left	Left	Bilateral	Left
Neurologic deficit(s)	Right hemiparesis	Right hemiparesis	Right hemiparesis, aphasia	Right hemiparesis
Neuroimaging	Cortical and white matter signal	Cortical and white matter signal	Cortical and white matter signal	Cortical and white matter signal
Surgery, age	Left hemispherectomy, 4 years	Left hemispherectomy, 11 years	SPT, 17 years; left face/hand area resection, 17 years	Left face/hand area resection, 18 years
Neuropathology	Mononuclear cell infiltration with gliosis	Mononuclear cell infiltration with gliosis	Mononuclear cell infiltration with gliosis	Mononuclear cell infiltration with gliosis
Clinical outcome	Engel class I, seizure-free (at 14 months)	Engel class I, seizure-free (at 24 months)	Engel class IV, death	Engel class III, 2 years (lost to follow-up)
Treatment(s)	GCs, no benefit	GCs, good response	GCs, IVIg, tacrolimus, cyclophosphamide (50 mg/kg)	GCs, IVIg, methotrexate, no benefit

CPS, complex partial seizure; EPC, epilepsia partialis continua; GCs, glucocorticoids; Gen, generalized seizures; IVIg, intravenous immune globulin; RE, Rasmussen’s encephalitis; SPT, subpial transaction.

### Neuropsychological studies

Neuropsychological assessments were conducted for clinical purposes of treatment and educational planning. The frequency of assessments varied among patients (patients 2, 3, and 4) depending on their clinical course, response to therapies, and medical interventions. However, for all cases the test battery included standard measures of intelligence (Wechsler Intelligence Scale for Children (WISC): WISC-III or WISC-IV), language (Boston Naming Test (BNT), Controlled Oral Word Association (COWA), animal naming), immediate and delayed verbal and visual memory (Wide Range Assessment Memory and Learning (WRAML) Story Memory and Design Memory, Rey Auditory Verbal Learning Test (AVLT), Rey-Osterrieth Complex Figure (ROCF)), motor skills (finger tapping, grooved pegboard), and processing speed (WISC coding/index, Trail Making Test (TMT) Part A). Serial neuropsychological examinations were performed at presentation and at subsequent time points (1 year after initial assessment for patients 2 and 4; 2 years after initial assessment for patient 3) as part of routine evaluation prior to surgery [16].

### Tissue samples

Frozen brain tissue (cortex and white matter) was collected from RE patients (n = 3, tissue was not available from patient 4) and control subjects undergoing cerebral resections for epilepsy (non-RE; n = 5; mean age 19 ± 10 years) for molecular and neuropathological studies, as described previously [17]. These studies were approved by the University of Alberta Ethics Committee (Pro00002291).

### Neuropathological studies

All patients underwent hemispherectomy or cortical resection and all tissues were reviewed at gross assessment and after histological and immunocytochemical staining by a certified neuropathologist (EJ) [17]. Next, 10 μM paraffin-embedded sections of human brain tissue from RE and non-RE were deparaffinized and hydrated using decreasing concentrations of ethanol. Sections were boiled in 0.01 M citrate buffer, pH 6.0, for 10 minutes prior to application of antibodies that detected CD3, CD8, CD68, vimentin, IL-1β, caspase-1, major histocompatibility complex (MHC) class II, and alanine/serine/cysteine (ASC); antibodies included anti-ASC (AL177, Adipogen, San Diego, CA, USA), anti-IL-1β and anti-caspase-1 (sc-7884 and sc-515, respectively, Santa Cruz Biotechnology, Dallas, TX, USA), anti-MHC class II (M0775, Dako, Glostrup, Denmark), anti-IL18 (D043-3, MBL International, Woburn, MA, USA), anti-CD3 (790–4341, Ventana, Tuscon, AZ, USA), anti-CD8 (M7103, Dako), anti-CD68 (M0814, Dako), and anti-vimentin (M0725, Dako). Primary antibodies were diluted in PBS/serum and incubated overnight at room temperature. Immunolabeling with primary antibodies was detected with biotinylated goat-anti-rabbit or biotinylated goat-anti-mouse (Vector Laboratories, Burlingame, CA, USA) and avidin-biotin-peroxidase complex (ABC, Vector Laboratories). Sections were counter-stained with hematoxylin.

### Human neural cell cultures

Human fetal astrocytes (HFAs), microglia, and neurons were prepared from 15- to 19-week fetal brains obtained, with consent (approved by the University of Alberta Ethics Committee, Pro00027660), as previously described [17-21]. Briefly, fetal brain tissues were dissected, meninges were removed and digested for 30 minutes with 2.5% trypsin (Life Technologies, Burlington, ON, Canada) and 2 mg/mL DNase I (Roche Diagnostics, Mannheim, Germany), and passaged through a 70 μm cell strainer (BD Biosciences, Mississauga, ON, Canada). Cells were washed twice with centrifugation at 14,000 rpm for 10 minutes and plated in T-75 flasks coated with poly-L-ornithine (Sigma-Aldrich, Oakville, ON, Canada) at 6 to 8 × 10^7^ cells/flask with media. Following a week of incubation (37°C at 5% CO_2_), adherent HFAs were separated from suspended microglia and re-plated for primary astrocytic, neuronal, or microglia differentiation (80% confluence). Cells were maintained in MEM containing 10% FBS (Life Technologies), 1% penicillin/streptomycin (Life Technologies), 1% L-glutamine (Life Technologies), and 10% dextrose (Life Technologies).

### Real-time RT-PCR

Tissues were homogenized and then lysed in TRIzol (Invitrogen, Carlsbad, CA, USA) and used in real-time PCR analyses as previously described. Briefly, human cerebral samples (white matter and adjacent cortex) and cultured cells were homogenized in TRIzol and total RNA was purified from the aqueous phase using RNeasy mini columns (Qiagen, Valencia, CA, USA) [16]. Following digestion of RNA with DNase I (Promega, Madison, WI, USA), cDNA was prepared by oligo(dT) primed reverse transcription of equal quantities of RNA using either SuperScript II or SuperScript III Reverse Transcriptase (Invitrogen) according to the manufacturer’s recommended protocols. Semi-quantitative real-time RT-PCR was performed using iQ SYBR Green supermix (Bio-Rad Laboratories, Hercules, CA, USA) on either an iQ5 or iCycler (Bio-Rad Laboratories) according to the manufacturer’s recommended protocols. Oligonucleotide PCR primers are provided in Table 2. All data were normalized to GAPDH mRNA levels and expressed as relative fold changes (RFCs) compared to non-RE controls.

**Table 2:** Oligonucleotide PCR primers

**Gene**	**Primer**
*ASC*	Forward 5′-GCC TGC ACT TTA TAG ACC AGC-3′
	Reverse 5′-GCT TCC GCA TCT TGC TTG G-3′
*CASP1*	Forward 5′-TCC AAT AAT GGA CAA GTC AAG CC-3′
	Reverse 5′-GCT GTA CCC CAG ATT TTG TAG CA-3′
*CD3e*	Forward 5′-GAT GCA GGG CAC TCA CT-3′
	Reverse 5′-CAT TAC CAT CTT GCC CCC AA-3′
*GAPDH*	Forward 5′-AGC CTT CTC CAT GGT GGT GAA GAC-3′
	Reverse 5′-CGG AGT CAA CGG ATT TGG TCG-3′
*HLA-DRA*	Forward 5′-GGA CAA AGC CAA CCT GGA AA-3′
	Reverse 5′-AGG ACG TTG GGC TCT CTC AG-3′
*IL1b*	Forward 5′-CCA AAG AAGAAG ATG GAA AAG C-3′
	Reverse 5′-GGT GCT GAT GTA CCA GTT GGG-3′
*IL18*	Forward 5′-TCT TCA TTG ACC AAG GAA ATC GG-3′
	Reverse 5′-TCC GGG GTG CAT TAT CTC TAC-3′
*NLRP1*	Forward 5′-ATT CCA GTT TGT GCG AAT CCA-3′
	Reverse 5′-GTT CCT TGG GGA GTA TTT CCA G-3′
*NLRP3*	Forward 5′-GAT CTT CGC TGC GAT CAA CAG-3′
	Reverse 5′-CGT GCA TTA TCT GAA CCC CAC-3′
*NLRC4*	Forward 5′-TGC ATC ATT GAA GGG GAA TCT G-3′
	Reverse 5′-GAT TGT GCC AGG TAT ATC CAG G-3′
*CD8*	Forward 5′-CCG ACG ATC ATG CAG AAG TA-3′
	Reverse 5′-GGT GAA GAG GTG GAA CAG GA-3′
*GFAP*	Forward 5′-GAG ATC GCCACC TAC AG-3′
	Reverse 5′-CAC ATC CTT GRG CTC CTG-3′

### In vitro activation and protein expression

To stimulate protein expression or release from primary microglia or astrocytes, cells isolated as described above were seeded at a density of 7 × 10^5^ cells/well and treated for 18 hours with either TNFα (50 ng/mL) or LPS (25 ng/mL). Following LPS priming, cells were treated for 2 hours with either ATP (500 μM) or MDP (500 ng/mL) for 6 hours to further trigger formation of inflammasome complexes. The caspase inhibitor, zVAD-fmk (100 μM), was used to inhibit these responses. Cell lysates from treated cells were collected in standard Laemmli sample loading buffer run on 12% gels and analyzed by western blot. The same antibodies used for immunohistochemistry were used for western blotting (see above). Alternatively, to measure release of IL-1β from stimulated cells, cell culture supernatants were collected and analyzed by enzyme-linked immunosorbent assay (ELISA; DY201, R&D Systems, Minneapolis, MN, USA).

### Statistical analyses

For determination of differences in neuropsychological performance and transcript levels, Mann**–**Whitney *U* or Student’s *t*-tests were performed using GraphPad Prism version 5 for OS X (GraphPad Software, San Diego, CA USA; http://www.graphpad.com). *P* values <0.05 were considered significant.

## Results

### Clinical and demographic features

Herein, four individuals were identified with clinical, imaging, and pathological features of RE, ranging in age from 3 to 14 years at the time of presentation (three females, one male), exhibiting different seizure types and outcomes (Table 1), and all of whom underwent surgery for removal of the epileptogenic regions. All patients exhibited neuropathological features characteristic of RE including astrogliosis, leukocyte infiltration, and neuronal injury.

### Neuroimaging and neuropsychological studies

**Figure 1:**
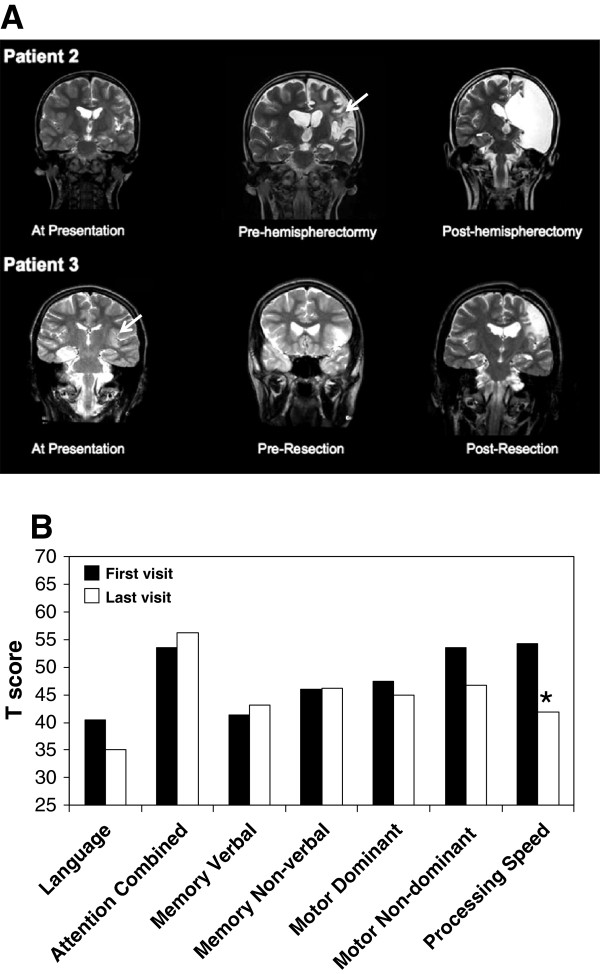
**Neuroimaging and neuropsychological profiles in Rasmussen’s encephalitis (RE). (A)** In patient 2, coronal MRI T2-weighted images at presentation revealed mild left hemisphere atrophy and cortical thinning, 3 years after presentation revealing profound left hemispheric atrophy and hyperintensity of white matter (arrowhead), and post-hemispherectomy. Similarly, in patient 3, increased white matter signal was evident at initial presentation but actually resolved after corticectomy. **(B)** Median T scores (population mean 50; standard deviation 10) in seven neuropsychological domains for patients 2, 3, and 4. Median first assessments are represented by black bars and the median last assessment as white bars. Mann–Whitney *U* test, *P <0.05. MRI, magnetic resonance imaging; RE, Rasmussen’s encephalitis.

Serial cranial MRI demonstrated increased signal apparent in both the cortex and adjacent matter in the affected (left) hemisphere (T2-weighted) for patient 2 (Figure 1A) with minimal gadolinium enhancement (data not shown). Similarly, patient 3 also exhibited increased signal in both cortex and white matter, which progressed over time and remained apparent after a partial resection of the lesion. Patients 1 and 4 demonstrated normal neuroimaging at presentation but developed significant atrophy of cortex and white matter of the left hemisphere within months of presentation (data not shown). Among patients 2, 3, and 4, longitudinal neuropsychological analyses were performed prior to surgery using established tests of language, attention, memory, motor functions, and processing speed. Median T scores (n = 3, patients 2 to 4) declined significantly between first and last assessments for processing speed, while memory (verbal and non-verbal), language, dominant motor, and non-dominant motor performances remained unchanged over time (Figure 1B). As expected with left hemispheric lesions, language function was reduced in all three patients at both early and late time points. Reliable neuropsychological assessment could not be performed in patient 1 due to the early age at presentation. The reduction in processing speed efficiency herein implied that subcortical structures might be affected, prompting examination of both cortex and adjacent white matter with molecular and morphological tools.

### Host neuroimmune responses

**Figure 2:**
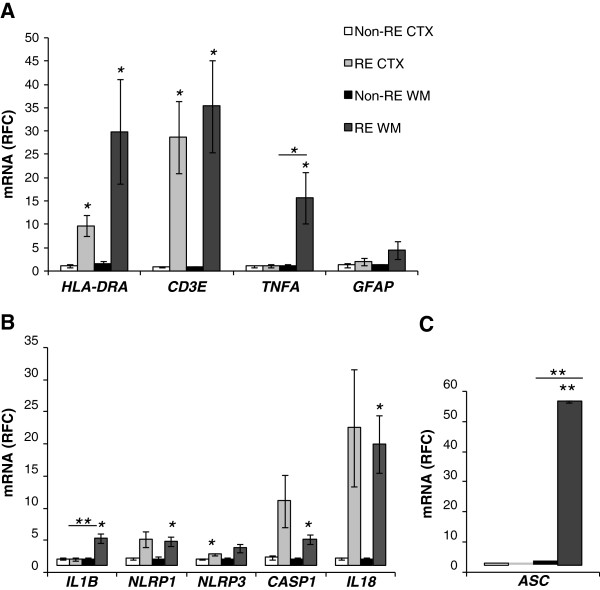
**Inflammatory gene expression in Rasmussen’s encephalitis (RE). A)** CD3ϵ, HLA-DR, TNFα and GFAP mRNA median RFC of white matter and cortical samples from RE patients (n = 3) compared to non-RE (n = 6) controls revealed significantly increased immune gene transcript levels in RE white matter samples compared to non-RE specimens. **B)** IL-1β, NLRP1, NLRP3, casp1, IL-18, and **C)** ASC mRNA RFC of white matter and cortical samples from RE patients compared to non-RE revealed an increase in expression of all genes in RE white matter as well as cortex for some genes. Student’s t-test, *P >0.05. RE, Rasmussen’s encephalitis; RFC, relative fold change.

Earlier studies indicate that leukocyte infiltration was a key aspect of the neuropathogenesis of RE [22,23], perhaps in response to expression of host autoantigens or infectious agents. Comparison of cortex and white matter transcript levels revealed that *HLA-DR* and *CD3ϵ* transcript levels were significantly increased in both cortex and adjacent white matter of RE patients (patients 1, 2, 3) compared to matched regions from non-RE patients (Figure 2A; Additional file 1). In addition, *TNF-α* transcript levels were induced in the white matter of RE patients compared to non-RE patients as well as matched cortical samples, while *GFAP* transcript levels were non-significantly increased in the white matter of RE patients (Figure 2A). Given the extent of neuroinflammation in the brain specimens of RE patients, particularly in white matter, the molecular machinery involved in inflammasome activation was investigated revealing that *IL-1β* transcript levels were increased in the white matter of RE patients compared to white matter from non-RE patients and matched RE cortical specimens (Figure 2B). Further analyses disclosed that *NLRP1* and *NLRP3* transcript levels were increased in RE white matter and cortex, while *casp1* and *IL-18* transcript levels were also significantly increased in both white matter and cortex of RE patients. *ASC* mRNA expression was selectively increased (by >80 fold) in white matter of RE patients (Figure 2B). These observations pointed to substantial increases in expression of genes implicated in inflammasome induction and function.

### Neural cell expression of inflammasome components

**Figure 3:**
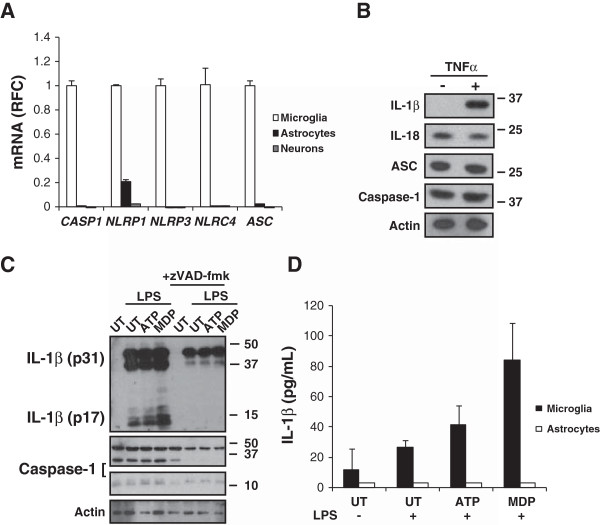
**Inflammasome expression in primary human neural cells. (A)** Immune gene transcript levels in primary human microglia, astrocytes, and neurons showing that microglia express all inflammasome-associated genes examined unlike astrocytes or neurons. **(B)** Expression of inflammasome components and substrates in primary microglia ± TNF-α (50 ng/mL). **(C)** IL-1β expression in LPS (25 ng/mL) primed microglia co-stimulated with ATP (500 M) or MDP (500 ng/mL) ± caspase inhibitor zVAD-fmk (100 M). **(D)** IL-1β release from LPS (25 ng/mL) primed microglia (but not astrocytes) co-stimulated with ATP (500 μM) or MDP (500 ng/mL). Student’s *t*-test, *P <0.05.

The above studies highlighted a role for inflammasome activation in RE but the cell type(s) contributing to increased inflammasome expression in the human nervous system remain uncertain. Analyses of mRNA from cultured primary human microglia, astrocytes, and neurons showed that *casp1*, *NLRP1*, *NLRP3*, *NLRC4*, and *ASC* transcripts were consistently detectable in human microglia (Figure 3A). In contrast, NLRP1 transcripts were expressed at low levels in human astrocytes, while the remaining genes were minimally detected in astrocytes and neurons. Thus, subsequent studies focused on primary human microglia. In order to examine expression of inflammasome components and substrates within microglia prior to inflammasome activation, lysates from untreated cells or cells stimulated with TNF-α were examined by western blot. Exposure of microglia to TNF-α induced pro-IL-1β immunoreactivity in cell lysates without altering the constitutive expression of IL-18, ASC, or caspase-1 (Figure 3B). To explore the induction of IL-1β in more depth, human microglia were primed with endotoxin (LPS) as a ‘signal 1’ followed by exposure to established activators of inflammasomes including ATP or MDP (‘signal 2’), revealing that under these conditions LPS alone not only induced expression of pro-IL-1β but also triggered its cleavage, yielding the mature form (IL-1β p17). However, this cleavage event was accentuated by exposure to the signal 2 molecules (Figure 3C). Treatment of stimulated microglia with the general caspase inhibitor, zVAD-fmk, eliminated the occurrence of mature IL-1β (Figure 3C). To investigate the release of IL-1β from stimulated glial cells, supernatants from LPS primed human microglia and astrocytes exposed to ATP or MDP showed detectable IL-1β in supernatants from microglia but without IL-1β release from astrocytes. These studies suggested the inflammasome components were principally located in microglia and that cultured human microglia could produce IL-1β with exposure to only a signal 1 but addition of a signal 2 enhanced the expression and release of IL-1β.

### Neuropathological studies

**Figure 4:**
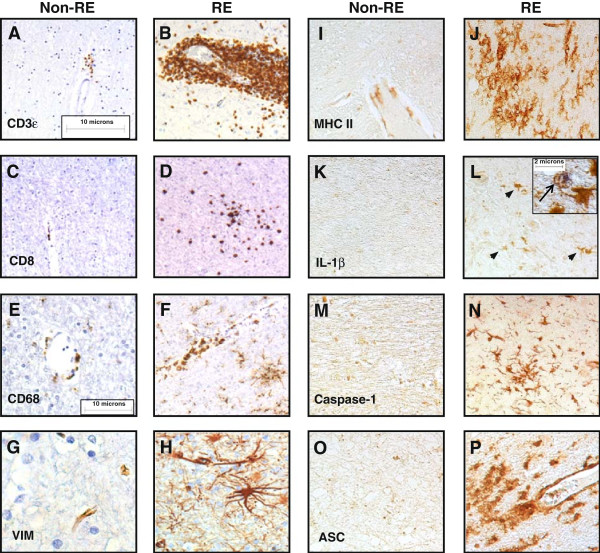
**Inflammatory proteins in Rasmussen’s encephalitis (RE).** Immunolabeling of CD3ϵ revealed minimal immunoreactivity in **(A)** the non-RE case and likewise **(C)** CD8, **(E)** CD68, and **(G)** vimentin immunoreactivity was infrequently detected in non-RE brain sections compared to **(B)** marked perivascular CD3ϵ-positive T cell abundance, numerous **(D)** CD8+ T cells, **(F)** CD68+ macrophages, and **(H)** hypertrophied astrocytes in the RE sections. Sections from the non-RE case showed negligible **(I)** MHC class II, **(K)** IL-1β, **(M)** caspase-1, and **(O)** ASC immunostaining, while **(J, L, N, P)** the RE white matter exhibited robust immunoreactivity for all proteins. IL-1β immunoreactivity was evident on cells resembling microglia in RE tissue (L, arrowheads); IL-1β (blue) was co-localized with MHC class II (brown) immunopositivity, as indicated by the arrow (L, inset). Original magnification: panels (**A,B,C,D)** x100, panels (E,F,G,H,I,J,K,L,M,N,O,P) x200; size bar 10 μm, inset x600; size bar 2 μm). ACS, alanine/serine/cysteine; MHC, major histocompatibility complex; RE, Rasmussen’s encephalitis.

Given the above clinical, imaging, neuropsychological, and molecular findings, we next examined the neuropathological features of RE. Analyses of white matter from non-RE and RE patients, disclosed CD3ϵ-immunopositive cells were highly abundant in perivascular spaces and parenchyma in RE brain sections (Figure 4B) compared to non-RE white matter (Figure 4A). CD8 immunopositive cells were infrequently observed in the non-RE white matter (Figure 4C) but were readily detected in RE white matter (Figure 4D). Occasional CD68+ perivascular myeloid cells were found in the non-RE white matter (Figure 4E), while numerous hypertrophied CD68+ cells resembling microglia and macrophages were evident in RE white matter including microglial nodules (Figure 4F). Vimentin immunoreactivity was apparent in endothelial cells in non-RE brains (Figure 4G) but was markedly increased in RE white matter in both endothelia and astrocytes (Figure 4H). Similar neuropathological changes were observed in the adjacent cortex from RE patients: immunolabeling disclosed minimal CD3ϵ (Additional file 2: Figure S2A), CD8 (Additional file 2: Figure S2B), and CD68 (Additional file 2: Figure S1C) in non-RE cortex compared with RE cortical sections (Additional file 2: Figure S2D,E,F). Thus, inflammation involving activated myeloid, lymphoid, and astrocytic cells was evident in both white matter and cortex in RE sections.

### Inflammasome expression in RE

To extend the findings of increased transcript expression of inflammasome-associated genes in the brains from RE patients (Figure 1), MHC class II immunoreactivity was investigated showing markedly increased expression in the white matter of RE brain tissues (Figure 4J) compared to non-RE patients (Figure 4I) with similar changes in the adjacent cortex (Additional file 3: Figure S3A). IL-1β immunoreactivity was not detected in white matter from non-RE patients (Figure 4K) but was apparent in RE white matter, localized in cells resembling microglia (Figure 4L), which was verified by co-localization with MHC class II immunoreactivity (Figure 4L, inset). IL-1β immunoreactivity was also detected in the microglial-like cells in RE cortex (Additional file 3: Figure S3B). Caspase-1 immunopositive cells, which also resembled microglia, were abundant in RE white matter (Figure 4N) in contrast to minimal detection in non-RE tissue (Figure 4M) with similar detection levels in RE cortex in microglia (Additional file 3: Figure S3D). In white matter from RE patients, ASC immunostaining was frequently detected in cells with morphological appearance of activated myeloid cells (Figure 4P) but was minimally found in non-RE white matter (Figure 4O). However, ASC immunopositive cells were scarce in RE cortex (Additional file 3: Figure S3D). These findings emphasized the induction of inflammasome components in RE brain tissues, particularly in white matter, in keeping with chronic inflammation and the associated neuroimaging and neuropsychological findings.

## Discussion

The present study represents the first analysis of RE patients integrating neuroimaging, neuropsychological, neuropathological, and molecular findings, which revealed both white matter and cortex involvement in the RE disease process with a concurrent induction of inflammasome components. This report is also one of the first to examine inflammasome expression and activation in human neural cells (microglia, astrocytes, neurons), disclosing that microglia and macrophages are the chief cells demonstrating inflammasome cellular machinery. There was evidence of lymphocyte invasion of RE brain tissues, particularly CD8+ T cells, in keeping with previous RE studies [8,22,23]. These immune perturbations likely contribute to the pathogenesis of RE, perhaps indicating an ability to respond by activating an inflammasome complex. The present observations also highlight the broader extent of cerebral tissue disruption (cortex and white matter) in RE, which was evident in multiple investigative platforms.

There has been a strong emphasis on the cerebral cortex as the principal target of tissue damage in RE, predicated largely on neuropathological studies showing gliosis and neuronal dysplasia and/or loss [22,24]. However, limited attention has been focused on pathological changes in adjacent white matter in RE, although several earlier studies show apparent changes in white matter in both neuropathological and neuroimaging analyses [10,22,25]. This study integrating neuroimaging, neuropsychological assessments, and expression of neuroinflammatory markers is consistent with progressive cerebral white matter involvement. The present serial neuropsychological assessments reveal current (progressive) white matter pathogenesis in RE because processing speed is dependent on white matter integrity; this variable worsened in the present cohort over time. It is unclear if white matter compromise early in the course of disease is reversible. Importantly, the finding of impaired language function early in the course of disease and early white matter changes on neuroimaging might implicate either early white matter disconnection and/or cortical injury [26].

The etiology of RE is unknown although both infectious and autoimmune etiologies have been postulated. The worldwide identification of RE makes an infectious agent a less plausible etiology unless it is a ubiquitous agent infecting a selectively ‘neurosusceptible’ group [27]. An autoimmune pathogenesis remains a strong possibility, as the current study revealed a combination of significant lymphocyte infiltration and gliosis in white matter suggesting immune dysregulation. Relevant to this finding, a study of CD8+ T cells in RE reveals the persistence of cytotoxic T lymphocytes in close proximity to neurons and astrocytes, consistent with antigen-driven CD8+ T cells mediating attack on neurons and astrocytes [8,23]. This current study shows for the first time that inflammasome activation is highest in human microglia and induction of the inflammasome results in selective release of IL-1b by microglia, but not astrocytes. This observation, coupled with our finding that genes involved in inflammasome activation are highest in the white matter of RE patients, along with the ubiquitous finding of microglial activation in RE supports a role for inflammasome activation in microglia in the pathogenesis of RE. Currently the role of the inflammasome in other central nervous system inflammatory disorders is unclear particularly in other white matter diseases such as multiple sclerosis, and warrants further investigation. Delineation of which inflammasome complex is activated in RE might lead to further insights into the etiopathogenesis of RE. Both NLRP1 and NLRP3 exhibited increased expression in RE brain specimens relative to controls with mesial temporal sclerosis and multiple sclerosis (Figure 1). However, it is plausible that other inflammasomes might exhibit increased expression. Future studies will need to examine newer inflammasome components as they become better defined but the present studies highlight the enhanced expression of both NLRP1 and NLRP3 in brains from RE patients. Although this study is limited by small numbers due primarily to the rarity of this disease, these findings raise the possibility of targeting inflammasomes as part of future therapeutic strategies for RE. Several possible agents include glyburide, which modulates pannexin-1, and new drugs such as cytokine release inhibitory drug (CRID)3 and milk fat globule-EGF factor 8 protein (MFGE8) [28,29].

## Conclusions

The significance of white matter neuroinflammation in terms of the etiology and pathogenesis for RE points to a more generalized cerebral disorder. The present findings, as well as previous studies are consistent with autoimmune or infectious etiologies for RE. Relevant to this point, current therapies consist of intravenous glucocorticoid therapy and eventual hemispherectomy although tailoring treatments to such a heterogeneous (and rare) patient population is challenging. There is a variable response to glucocorticoids early in RE, but eventually most patients relapse; in bilateral hemispheric disease or late-onset dominant hemisphere disease functional hemispherectomy is not a feasible option. Moreover, this cohort received several broad immunosuppressive agents including one patient with immunoablative cyclophosphamide treatment without discernible effects on disease progression, suggesting that novel targeted therapies for RE are urgently required. The findings also imply that targeting of the inflammasomes might represent a novel therapeutic strategy, warranting multicenter validation in larger cohorts of patients with RE.

## Abbreviations

ABC: Avidin-biotin-peroxidase complex; ASC: Alanine/serine/cysteine; AVLT: Auditory verbal learning test; BNT: Boston naming test; COWA: Controlled oral word association; CRID: Cytokine release inhibitory drug; ELISA: Enzyme-linked immunosorbent assay; FBS: Fetal bovine serum; GAPDH: Glyceraldehyde 3-phosphate dehydrogenase; H&E: Hematoxylin and eosin; HFA: Human fetal astrocyte; IL: Interleukin; LPS: Lipopolysaccharide; MDP: Muramyl dipeptide; MEM: Minimal Essential Medium; MFGE8: Milk fat globule-EGF factor 8 protein; MHC: Major histocompatibility complex; MRI: Magnetic resonance imaging; PBS: Phosphate buffered saline; PCR: Polymerase chain reaction; RE: Rasmussen’s encephalitis; RFC: Relative fold change; ROCF: Rey-osterrieth complex Figure; RT: Reverse transcriptase; TMT: Trail making test; TNF: Tumor necrosis factor; WISC: Wechsler intelligence scale for children; WRAML: Wide range assessment memory and learning.

## Competing interests

The authors declare that they have no competing interests.

## Authors’ contributions

VR, DBS, DWG, and CP conceived the project. VR performed real-time PCR studies, analyzed and interpreted data, and participated in overall study design. WB and FM performed the real-time studies, analyzed and interpreted the data. JGW performed and interpreted the *in vitro* experiments. TJS performed, analyzed, and interpreted the neuropsychological assessments. EJ interpreted the neuropathological findings. FM and WB performed and analyzed immunohistochemical staining. DBS, RTW, BM, and DWG collected, analyzed, and interpreted results. CP supervised all aspects of the study. VR and CP wrote the manuscript. All authors read and approved the final version of the manuscript.

## Supplementary Material

Additional file 1: Figure S1 CD3ϵ and HLA-DRA expression in cortex and white matter of patient 3 comparing right and left hemispheres revealed markedly increased expression in white matter bilaterally with minimal gene expression changes in cortex. All values were normalized to GAPDH expression. Click here for file

Additional file 2: Figure S2 Cortical changes in RE. **(A)** CD3ϵ expression in cortex of non-RE patient compared with **(B)** RE. H&E staining shows cortical injury in **(D)** RE cortex (i, arrow in inset) and CD8 immunoreactivity in **(B)** non-RE cortex compared with **(E)** RE cortex. CD68 immunolabeling of macrophages in **(C)** non-RE and **(F)** RE cortex. Original magnification: panels (A,B,D,E) x100, panels (C,F) x200. Click here for file

Additional file 3: Figure S3 Cortical changes in RE. **(A)** MHC class II expression in cortex of non-RE patient compared with **(E)** RE. IL-1β immunoreactivity in **(B)** non-RE cortex compared to **(F)** RE cortex. Caspase-1 immunoreactivity in **(C)** non-RE cortex compared with **(G)** RE cortex. ASC immunolabeling of macrophages in **(D)** non-RE and **(H)** RE cortex. Original magnification x200. Click here for file
