# Supplementary material for: Inflammasome induction in Rasmussen’s encephalitis: cortical and associated white matter pathogenesis
Source: J Neuroinflammation. 2013 Dec 13;10:152. doi: 10.1186/1742-2094-10-152 (PMC3881507; doi:10.1186/1742-2094-10-152)
Supplement: Additional file 1: Figure S1 — CD3ϵ and HLA-DRA expression in cortex and white matter of patient 3 comparing right and left hemispheres revealed markedly increased expression in white matter bilaterally with minimal gene expression changes in cortex. All values were normalized to GAPDH expression. [file 1742-2094-10-152-S1.pdf]

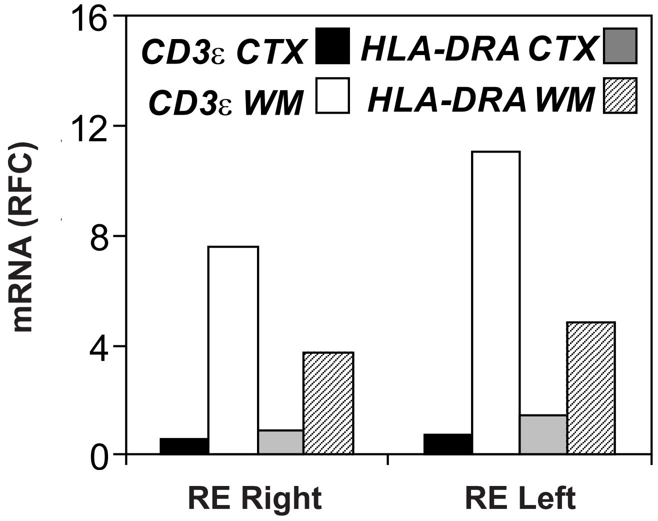

Supplementary Figure 1: CD3ε and HLA-DRA expression in cortex and white matter of Patient 3 comparing right and left hemispheres revealed markedly increased expression in white matter bilaterally with minimal gene expression changes in cortex. All values were normalized to GAPDH expression.
