# Supplementary material for: Inflammasome induction in Rasmussen’s encephalitis: cortical and associated white matter pathogenesis
Source: J Neuroinflammation. 2013 Dec 13;10:152. doi: 10.1186/1742-2094-10-152 (PMC3881507; doi:10.1186/1742-2094-10-152)
Supplement: Additional file 2: Figure S2 — Cortical changes in RE. (A) CD3ϵ expression in cortex of non-RE patient compared with (B) RE. H&E staining shows cortical injury in (D) RE cortex (i, arrow in inset) and CD8 immunoreactivity in (B) non-RE cortex compared with (E) RE cortex. CD68 immunolabeling of macrophages in (C) non-RE and (F) RE cortex. Original magnification: panels (A,B,D,E) x100, panels (C,F) x200. [file 1742-2094-10-152-S2.pdf]

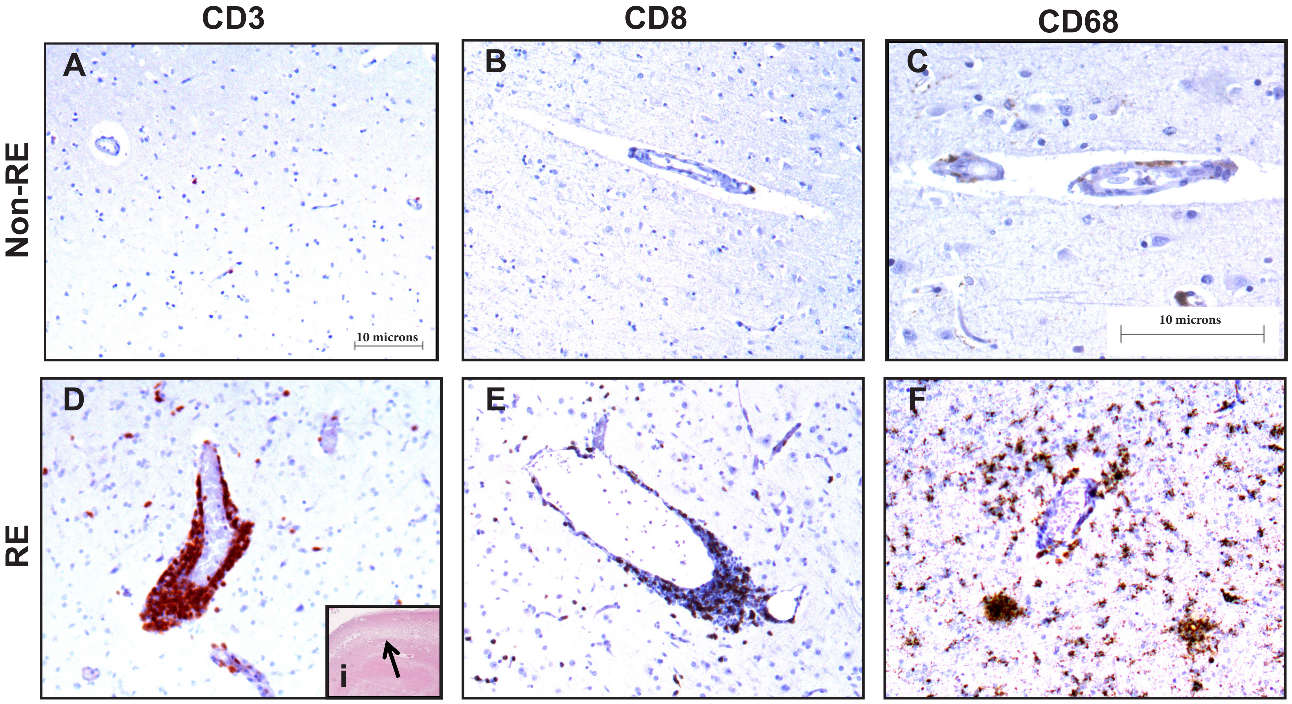

Supplementary Figure 2: Cortical changes in RE. (A) CD3ε expression in cortex of non-RE patient compared with RE (B). H&E staining shows cortical injury in RE cortex (Di, arrow in inset) CD8 immunoreactivity in non- RE cortex (B) compared with RE cortex (E). CD68 immunolabeling of macrophages in non-RE (C) and RE (F) cortex. (riginal magnification A,B,D and E 100X, C and F 200X)
