# Supplementary material for: Inflammasome induction in Rasmussen’s encephalitis: cortical and associated white matter pathogenesis
Source: J Neuroinflammation. 2013 Dec 13;10:152. doi: 10.1186/1742-2094-10-152 (PMC3881507; doi:10.1186/1742-2094-10-152)
Supplement: Additional file 3: Figure S3 — Cortical changes in RE. (A) MHC class II expression in cortex of non-RE patient compared with (E) RE. IL-1β immunoreactivity in (B) non-RE cortex compared to (F) RE cortex. Caspase-1 immunoreactivity in (C) non-RE cortex compared with (G) RE cortex. ASC immunolabeling of macrophages in (D) non-RE and (H) RE cortex. Original magnification x200. [file 1742-2094-10-152-S3.pdf]

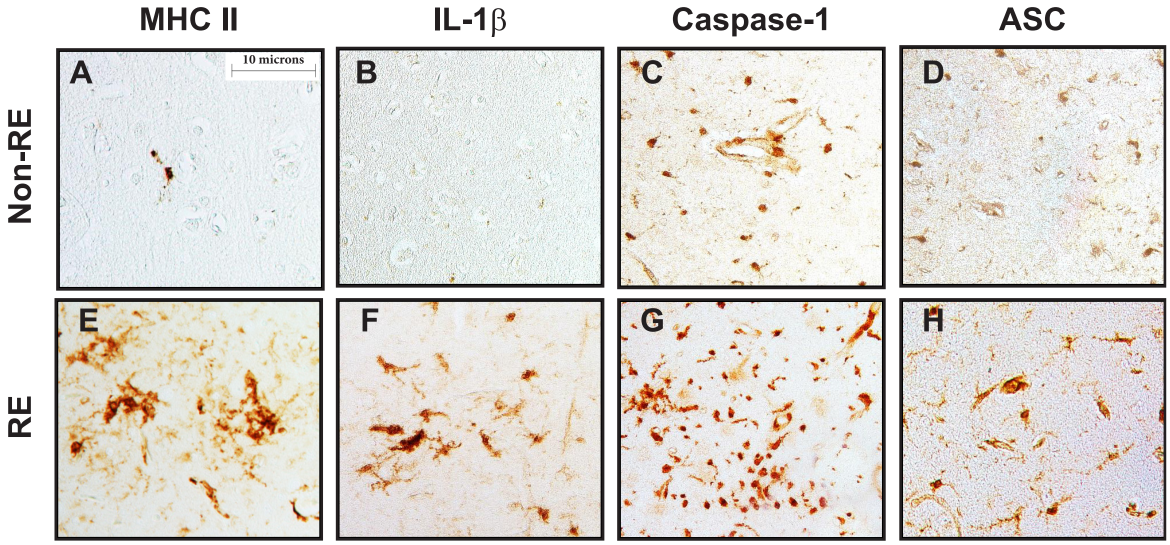

Supplementary Figure 3: Cortical changes in RE. (A) MHC Class II expression in cortex of non-RE patient compared with RE (E). IL-1β immunoreactivity in non-RE cortex (B) compared to RE cortex (F). Caspase-1 immunoreactivity in non- RE cortex (c) compared with RE cortex (G). ASC immunolabeling of macrophages in non-RE (D) and RE (H) cortex. (Original magnification 200X)
